# Effects of Glucose Control and Variability on Endothelial Function and Repair in Adolescents with Type 1 Diabetes

**DOI:** 10.1155/2013/876547

**Published:** 2013-12-29

**Authors:** Robert P. Hoffman, Amanda S. Dye, Hong Huang, John A. Bauer

**Affiliations:** ^1^Department of Pediatrics, The Ohio State University, The Research Institute at Nationwide Children's Hospital, 700 Children's Drive, ED422, Columbus, OH 43205, USA; ^2^Department of Pediatrics, West Virginia University, 830 Pennsylvania Avenue Suite 104, Charleston, WV 25302, USA; ^3^Research Institute at Nationwide Children's Hospital, 700 Children's Drive, Columbus, OH 43205, USA

## Abstract

*Background*. Endothelial dysfunction and increased inflammation are precursors of cardiovascular disease in type 1 diabetes (T1D) and occur even in adolescents with T1D. The goal of this study was to determine the relationship of endothelial dysfunction to various measures of glycemia. *Research Design and Methods*. Forearm blood flow (FBF, venous occlusion plethysmography) was measured before and after 5 min of upper arm vascular occlusion in 17 adolescents with uncomplicated type 1 diabetes. Endothelial function was assessed as postocclusion FBF and forearm vascular resistance (FVR, mean arterial pressure/FBF). Fasting glucose, 72 hour mean glucose and standard deviation from continuous glucose monitoring, hemoglobin A1c, and hemoglobin A1c by duration area under the curve were used to assess immediate, short-term, and intermediate- and long-term glycemia. *Results*. Postocclusion FBF (*r* = −0.53, *P* = 0.030) negatively correlated and postocclusion FVR positively correlated (*r* = 0.52, *P* = 0.031) with hemoglobin A1c levels. FVR was positively associated with log 3 day mean glucose (*r* = 0.55, *P* = 0.027). Postocclusion FBF (2.8 ± 1.1 versus 3.4 ± 0.5 mL/dL/min, mean ± SE, *P* = 0.084) tended to be lower and FVR (31.4 ± 10.4 versus 23.9 ± 4.4 mmHg dL min/mL, *P* = 0.015) was significantly higher in subjects with hemoglobin A1c above the median (8.3%) compared to those with lower hemoglobin A1c levels. *Conclusions*. These results demonstrate that poor intermediate-term glycemic control is associated with impaired endothelial function.

## 1. Background

Vascular endothelial dysfunction contributes to the development of both macrovascular and microvascular complications in type 1 diabetes (T1D) and there is considerable evidence that patients with T1D have increased endothelial stress/injury and reduced endothelial repair. Recent studies have indicated that adolescents with T1D have impaired flow mediated vasodilation, as measured using either brachial artery ultrasound or venous occlusion plethysmography [1–6]. Flow mediated vasodilation is due to endothelial nitric oxide release and thus is an effective measure of endothelial function.

Beyond this, there is good evidence of impaired endothelial repair capacity in T1D. Studies in mice indicate that animals with streptozotocin-induced diabetes have impaired vascular repair following hindlimb ischemia [7]. Similarly, vascular repair is impaired in control animals following transplantation of bone marrow derived stem cells from diabetic animals compared to those receiving cells from healthy animals [8]. Both studies indicated that progenitor cell counts were reduced in diabetic animals. Studies in humans have generally indicated reduced progenitors cell counts in young adults with T1D without overt complications [9–11]. Interestingly, in T1D adolescents, Dimeglio et al. [12] found decreased CD 34+CD133+CD31+ or circulating endothelial progenitor cells (CPCs), as reported in adults, but increased CD34+ CD45− cells or endothelial colony forming cells (ECFCs). They also found a negative relationship between the ECFCs and CPCs. They believed that the decreased CPCs indicated reduced repair capacity and the increased ECFCs were due to increased recruitment due to ongoing endothelial damage. The former conclusion was confirmed by a positive relationship between CPCs and endothelial function as measured using skin blood flow response to acetylcholine iontophoresis. Importantly, they found no relationships between any measure of glucose control and either CPCs or ECFCs.

Hyperglycemia-induced oxidative stress plays a key pathophysiological role in damaging endothelial function in diabetes [13, 14]. Acute hyperglycemia, also, profoundly increases muscle blood flow probably through osmotic effects in both healthy adults [15] and adolescents with T1D [16]. This pronounced increase in blood flow leads to a diminished incremental response to flow mediated vasodilation, possibly through a ceiling effect. A direct adverse effect of glucose variability on endothelial cells has, also, been demonstrated. Protein kinase-c levels, apoptotic rates, and biochemical markers of endothelial cell dysfunction are higher in endothelial cells exposed to intermittent glycemic levels of 90 or 360 mg/dL than in cells exposed continuously to either 90 or 360 mg/dL [17]. It is thought that the glucose fluctuations prevent the cells from adapting to the hyperglycemic condition. The adverse effect of oscillating glucose levels has been confirmed in adults with and without type 2 diabetes. Oscillating hyperglycemic and euglycemic clamp over 24 hours leads to greater impairment in endothelial function (measured using flow mediated vasodilation) and greater oxidative damage via reactive nitrogen species than does constant hyperglycemia [18].

Because of the above data, the primary goals of this study were to determine the effects of various measures of glycemia on endothelial function and endothelial repair capacity in adolescents with T1D. Specific measures that were studied include fasting glucose, 3 day average glucose and glucose standard deviation as determined via continuous glucose monitoring, hemoglobin A1c, and hemoglobin A1c area under the curve since diagnosis of diabetes (HbA1cAUC). Increasing our understanding how glucose control affects endothelial function and repair in adolescents will enhance our knowledge of the early pathophysiology of cardiovascular disease in T1D.

## 2. Methods

### 2.1. Subjects

17 adolescents (8 females, 9 males) with type 1 DM were recruited from the Pediatric Diabetes Clinic of Nationwide Children's Hospital (NCH). Their mean age was 13.1 ± 1.6 years (mean ± SD) and their mean body mass index was 20.3 ± 3.1 kg/m^2^. Mean HgbA1c was 8.3 ± 1.2% and mean duration of diabetes was 4.8 ± 3.8 years. The study was approved by the NCH Institutional Review Board and informed consent was obtained from parent or legal guardian. Proper assent was obtained from all subjects.

Screening included a history, physical exam, Tanner staging, and fasting laboratory testing. Type 1 DM was defined by the American Diabetes Association Criteria plus a fasting C-peptide of less than 0.4 ng/mL, insulin monotherapy since diagnosis, and an absence of a history of oral hypoglycemic agents and acanthosis nigricans on exam. All subjects were nonsmokers by report.

All subjects were Tanner stage 2–4 in order to minimize the effects of starting or finishing puberty. In order to limit confounding effects on endothelial function, subjects with BP > 95% tile, smoking, pregnancy, and uncorrected hypothyroidism were excluded. Subjects with microalbuminuria, overt nephropathy, or early renal failure (random urine microalbumin/creatinine > 0.02 mg albumin/mg creatinine; serum creatinine > 1.0 mg/dL) were also excluded.

### 2.2. Protocol

Subjects were admitted to the Clinical Research Center of the Wexner Medical Center at the Ohio State University at 0730 after having fasted from 2200 the night before. Subjects were instructed to take their usual basal insulin the night before or to remain on their usual basal insulin infusion rates if using continuous subcutaneous insulin. Subjects withheld their morning insulin bolus until breakfast was given after the study completion. Endothelial testing was then conducted as described below. A 20-minute rest period was held before endothelial-independent vasodilation assessed using sublingual 0.3 mg of glycerol trinitrate. A blood sample was then collected for measurement of fasting glucose and ECFCs. Eleven patients then participated in an insulin clamp study to assess the effects of glucose normalization and hyperglycemia. Results on this study have been previously reported [16]. Subjects were then placed on a continuous 24-hour glucose monitor (Medtronic Guardian, Fridley MN) for 3 days after which the monitor was returned and the data was downloaded using the Medtronic Carelink website to determine the mean glucose level and standard deviation.

### 2.3. Assessment of Endothelial and Nonendothelial Vasodilation

Forearm Blood Flow (FBF) was measured using strain gauge venous occlusion plethysmography, as previously described by Higashi and Yoshizumi [19], using a Hokanson EC6 plethysmograph (DE Hokanson Inc, Bellevue, WA) in the dominant arm. An indium-in-silastic strain gauge was attached to the widest portion of the forearm and connected to a plethysmography device. Sphygomanometric cuffs were placed on the arm at the wrist and on the upper arm. The wrist cuff was inflated to 200 mmHg to occlude blood flow to the hand for the duration of the study. During FBF measurement the upper arm cuff was inflated to 40 mmHg for 10 out of 15 seconds to occlude venous return but not arterial inflow. Each subject had two minutes of baseline flow recorded and then the upper arm cuff was inflated to 200 mmHg pressure for five minutes to occlude arterial flow to the arm. It was then released to create a sudden shear stress. FBF was again measured for the next minute. The FBF outflow signal was transmitted to a recorder (Powerlab 8, ADInstruments, Colorado Springs, CO) and FBF was expressed as mL per minute per 100 mL of forearm tissue volume. Forearm vascular resistance (FVR) was calculated by dividing mean arterial pressure (measured by automated sphygmomanometer) by FBF. All studies were scored by a single experienced investigator (RPH).

FBF was measured for 1 minute before and 5 minutes after glycerol trinitrate. The highest percent increase in 1 min postnitrate FBF or largest percent fall in FVR were used to assess endothelial-independent vasodilation.

### 2.4. Laboratory Measurements

Hemoglobin A1c levels were taken from the Pediatric Diabetes Clinic at Nationwide Children's. Measurements in the clinic are done using point of care testing on the DCA 2000 (Leverkusen, Germany).

ECFCs were measured using polychromatic flow cytometry methods. A 50 *μ*L volume anticoagulated peripheral blood was incubated with 50 *μ*L 3% BSA in PBS (without Ca++ and Mg++) at room temperature for 30 min. In dark, fluorescence labeled antibodies (2.5 *μ*L of each), PE-AC133, FITC-CD34, and PECy5-CD45, were added and incubated for 30 min at room temperature. FACS lysis buffer (450 *μ*L) was then added and incubated for 30 min at room temperature in dark. Samples were then analyzed on a FACS Calibur flow cytometer, where total counts are >400,000 cells. Intra-assay variability from ~100 *μ*L whole blood was <5%.

### 2.5. Statistical Analysis

HbA1cAUC was calculated using the trapezoidal rule. Pearson regression analysis was used to assess the relationship between vascular, ECFC's, and inflammatory and oxidative markers and the various measures of glucose control. *t*-tests were used for group comparisons. Data was log normalized, as needed. Analysis was performed using Systat 11 (SAS, Systat Software Inc, Chicago, IL). Results are shown as mean ± SD.

## 3. Results

### 3.1. Vascular Measures

**Figure 1:**
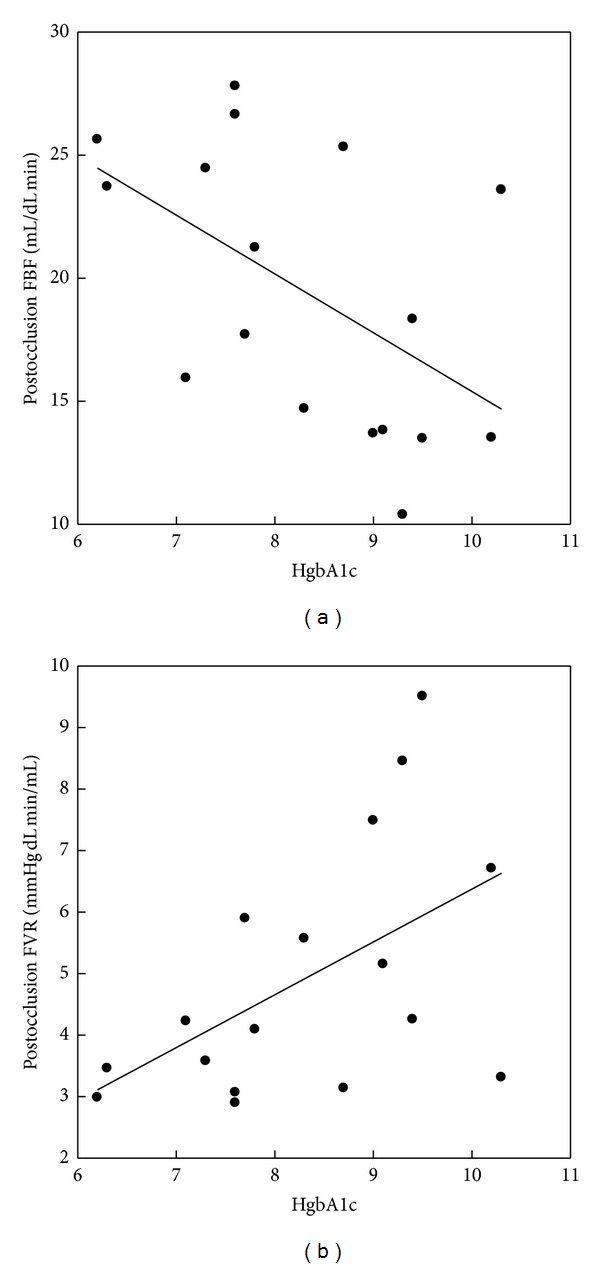
Relationships of postocclusion forearm blood flow (*r* = −0.53, *P* = 0.030) and vascular resistance to hemoglobin A1c in adolescents with type 1 diabetes (*r* = 0.52, *P* = 0.031).

Mean postocclusion FBF for the group was 19.2 ± 5.7 mL/dL min. Postocclusion FBF decreased as age increased (*r* = −0.55, *P* = 0.023) and as glucose control worsened as indicated by a negative relationship with the hemoglobin A1c at the time of study (*r* = −0.53, *P* = 0.030, Figure 1). The diminished postocclusive FBF was due to impaired vasodilation since postocclusive FVR increased as single hemoglobin A1c increased (*r* = 0.52, *P* = 0.031). Postocclusive FBF was not related to fasting glucose, 3 day mean glucose, or HbA1cAUC.

**Figure 2:**
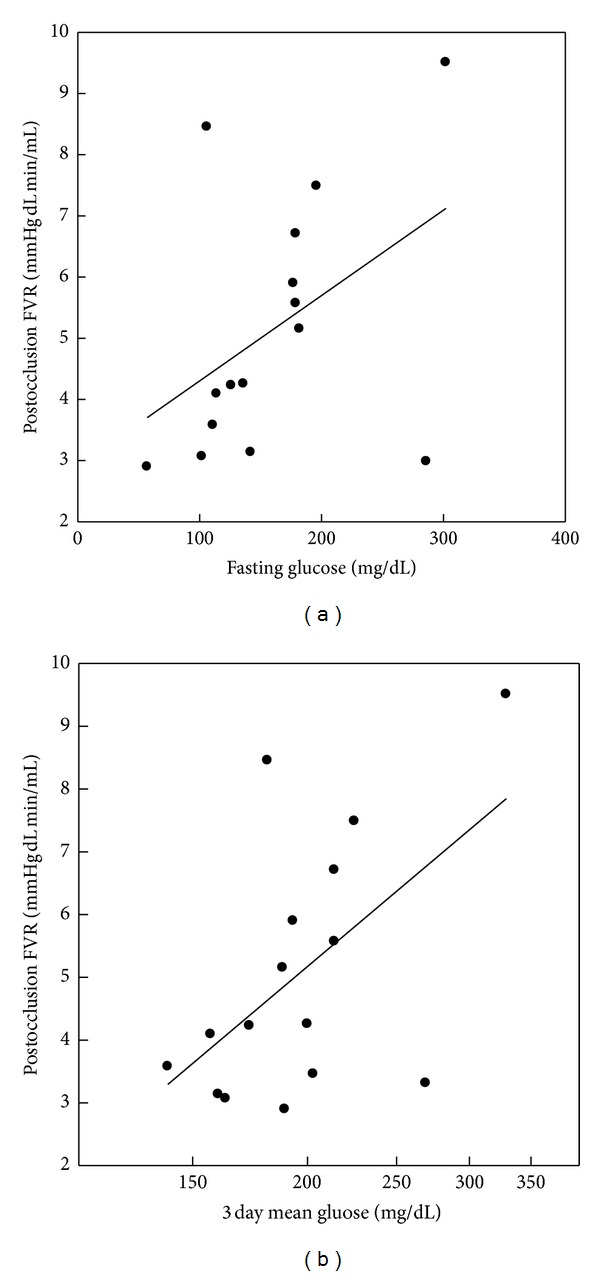
Relationships of postocclusion forearm vascular resistance to fasting glucose (*r* = 0.44, *P* = 0.098) and 72 hour mean glucose (*r* = 0.55, *P* = 0.027) in adolescents with type 1 diabetes.

Mean postocclusive FVR was 4.9 ± 2.0 mmHg dL min/mL. Postocclusive FVR was positively related to log 3 day mean glucose (*r* = 0.55, *P* = 0.027Figure 2) and tended to be positively related to fasting glucose (*r* = 0.44, *P* = 0.098) but not to glucose standard deviation. Stepwise multiple regression including hemoglobin A1c, fasting, and log 3 day mean glucose revealed that only the log 3 day mean glucose significantly predicted postocclusion FVR. The ratio of post to preocclusive FBF was not related to any measure of glucose control while the percent change in FVR from preocclusion to postocclusion tended to worsen as fasting glucose increased (*r* = 0.46, *P* = 0.088).

Postocclusive FBF decreased as age increased (*r* = −0.55, *P* = 0.023) but was not related to BMI. None of the postocclusive FVR measures were significantly related to age or BMI. Neither postocclusive FBF nor postocclusive FVR was related to endothelial-independent vasodilation. Log absolute ECFC and percent ECFC were not significantly related to any glycemic measures, age, or BMI.

Median hemoglobin A1c for the 17 T1D subjects was 8.3%. To confirm the relationships of endothelial function to glycemic control, subjects were divided into two groups with hemoglobin A1c levels above and below the median.  Table 1 shows age, duration of diabetes, BMI, blood pressure, and pre- and postocclusion FBF and FVR values for the two groups. Age, duration of diabetes, BMI, and blood pressure did not differ between the two groups. Both pre- and postocclusion FVR were increased in subjects with poorly controlled compared to well-controlled T1D. The results were confirmed by the FBF results where postocclusion FBF tended to be lower in poorly controlled T1D compared to well-controlled T1D (*P* = 0.084). ECFC's and maximal vasodilation in response to sublingual nitroglycerin T1D did not differ between poorly and well-controlled T1D subjects.

## 4. Discussion

The results clearly demonstrate that in adolescent T1D poor intermediate-term glucose control impairs endothelial function since postocclusive FBF decreased and postocclusive FVR increased as hemoglobin A1c increased, indicating impaired maximal sheer stress and nitric oxide-induced vasodilation. This was confirmed by the increased postocclusive FVR and trend toward decreased FBF in poorly controlled T1D compared to well-controlled subjects.

Multiple methods have been used to assess endothelial function in the normal population and in adolescents with T1D [1–6]. These methods generally assess the change in either brachial artery diameter as indicated by ultrasound or the change in FBF or FVR as measured by venous occlusion plethysmography following upper arm vascular occlusion. While it is clear that this reactive hyperemic response is mediated by endothelial nitric oxide release [19], it is less clear which method is best to assess endothelial function in T1D. Furthermore, data from our laboratory indicates that acute hyperglycemia markedly increases preocclusion FBF and decreases preocclusion FVR which creates a ceiling and a floor effect, respectively, for assessing postocclusion changes in FBF and FVR [16]. Studies of young adult patients with T1D have demonstrated decreased basal limb blood flow in the leg [20] while studies in long standing T1D without complications have demonstrated increased limb blood flow in the arm [21]. Babar et al. found larger brachial artery baseline diameters in prepubertal subjects with T1D compared to control subjects [6]. From this data, it is clear that different plasma glucose levels may lead to alterations in basal (preocclusion) blood flow and brachial artery diameter that may potentially alter change measurements in FBF or brachial artery diameter, as indicators of endothelial function.

Recently, Nadeau et al. [4] have simply reported decreased postocclusive FBF in adolescents with T1D without assessing change scores. The diminished postocclusive FBF is likely an indicator of impaired, maximal endothelially induced vasodilation in adolescents with T1D. The data in the current study expand this finding by indicating that maximal postocclusive vasodilation decreases as intermediate- and short-term glucose control worsen as indicated by lower FBF and higher FVR with increasing hemoglobin A1c. The short-term nature of this effect was demonstrated by the fact that postocclusive FVR increased as 3 day average glucose increased. This decreased ability to increase blood flow in response to shear stress with poor glucose control may play a significant role in the development of cardiovascular disease in T1D. Endothelial dysfunction is, also, thought to play a significant role in the diabetic microvascular complications as well [13, 14, 22, 23].

In our study, intermediate-term and short-term glucose control (single hemoglobin A1c and 3 day mean glucose) were clearly associated with impaired endothelial function. This is the opposite of the findings of Babar et al. who found better endothelial function in T1D prepubertal subjects with poor glucose control [6]. They had no explanation for this finding. In older adolescents with less than 5 years duration, Ce et al. found that brachial artery flow mediated dilation significantly negatively correlated with mean hemoglobin A1c levels between 12 and 24 months before the measurement but not mean hemoglobin A1c levels between 0 and 12 months before the measurement [24]. They did not find this relationship in subjects with more than 5 years duration. They suggested that this relationship was an evidence of a metabolic memory affect. Our data is somewhat different in that we found a direct relationship with current hemoglobin A1c and no relationship to long-term glycemic control as indicated by HbA1cAUC. When only patients under 5 years duration were studied the relationships between FBF and single hemoglobin A1c and between FVR and log 3 day mean glucose remained significant (data not shown). Too few subjects had duration of more than 5 years to adequately assess whether there were duration related differences in these relationships but multilinear regression analysis did not reveal significant duration by hemoglobin A1c interactions. The reason that recent hemoglobin A1c had an effect in our study, and not theirs, is not immediately apparent. Possible explanations would include the younger subjects in the current study and different methods of assessing endothelial function. The importance of the effect of poor glycemic control is emphasized when postocclusion FBF and FVR from the T1D subjects are compared to that of results from healthy, pubertal control subjects from a previous study (FBF: 22.5 ± 4.5 mL/dL min; FVR: 4.1 ± 1.5 mmHg dL min/mL) [25]. Postocclusion FBF was significantly lower and FVR significantly increased in the subjects with hemoglobin A1c above the median.

Glucose variability was not significantly related to either postocclusive FBF or FVR. This finding was somewhat surprising since in vitro [17] and in vivo [18] studies have indicated that oscillating glucose leads to impaired endothelial function. These results, however, confirm results from Pena et al. which found no relationship between endothelial function, measured using brachial artery, flow-mediated vasodilation, and glucose variability from the continuous glucose monitor [5]. Interestingly, they did find a negative effect of hypoglycemia on endothelial function. We did not assess this relationship. Long-term glucose control was not significantly related to measures of endothelial function.

We found no relationships between ECFC's and glucose control. ECFC% was increased in our subjects compared to age-matched control subjects from a previous study (T1D, 0.115 ± 0.074 versus controls, 0.022 ± 0.017%, mean ± SD, *P* = 0.016) confirming previous findings by Dimeglio et al. [12]. They believed that the increased ECFCs in T1D were due to an ongoing response to endothelial injury; thus, it would be expected that ECFC counts would increase as endothelial function deteriorates. However, neither Dimeglio et al. nor we found a significant relationship between ECFCs and endothelial function [12]. They did find a negative relationship between CPCs, which we did not assess, and endothelial function. Hortenhuber et al. [26] found no difference in what they called circulating progenitor cells (CD 34+ CD133+) between control and type 1 diabetic subjects and lower endothelial progenitor cells (CD34+ CD133+ CD309+) in type 1 diabetes adolescents. They also found that these latter cells decreased as hemoglobin A1c increased.

The main limitation of this study is the small number of subjects studied which decreases our ability to truly assess the interactions between immediate, short-term, intermediate-term, and long-term glucose control and endothelial function. This was particularly true regarding postocclusive FVR which was significantly related to log 3 day mean glucose and single hemoglobin A1c and tended to be related to fasting glucose since log average glucose was positively correlated with both hemoglobin A1c (*r* = 0.56, *P* = 0.025) and fasting glucose (*r* = 0.88, *P* < 0.001) while the latter two were not related. The fact that postocclusive FBF was only related to single hemoglobin A1c supports our contention that intermediate glycemic control is most important.

## 5. Conclusions

Our results demonstrate that short and intermediate-term, poor glucose control are associated with diminished endothelial function in pubertal T1D adolescents. Since impaired endothelial function is a precursor for future cardiovascular disease, these results emphasize the need to achieve good diabetes control in adolescents.

## Figures and Tables

**Table 1:** Demographics, blood pressure, and pre-and postocclusion FBF and FVR in adolescents with type 1 diabetes according to glucose control.

	A1c ≤ 8.3%	A1c > 8.3%
Age (years)	12.5 ± 1.6	13.8 ± 1.2
Duration (years)	4.1 ± 2.5	5.4 ± 5.1
BMI (kg/m^2^)	20.1 ± 4.0	20.4 ± 2.0
SBP (mmHg)	106 ± 5	107 ± 7
DBP (mmHg)	63 ± 10	60 ± 8

	Preocclusion	
FBF (mL/dL min)	3.4 ± 0.5	2.8 ± 1.1
FVR (mmHg dL min/mL)	23.9 ± 4.4	31.4 ± 10.4^b^

	Postocclusion	
FBF (mL/dL min)	22.0 ± 4.8	16.5 ± 5.3^a^
FVR (mmHg dL min/mL)	4.0 ± 1.1	6.0 ± 2.3^c^

^a^ *P* = 0.084 versus well-controlled, ^b^ *P* = 0.047 versus well-controlled, and ^c^ *P* = 0.015 versus well-controlled.

## Abbreviations

BMI: Body mass index
CPCs: Circulating progenitor cells
ECFCs: Endothelial colony forming cells
FBF: Forearm blood flow
FVR: Forearm vascular resistance
HbA1cAUC: Area under curve of hemoglobin A1c by duration curve
T1D: Type 1 diabetes.

## References

[1] Donaghue KC, Robinson J, McCredie R, Fung A, Silink M, Celermajer DS (1997). Large vessel dysfunction in diabetic adolescents and its relationship to small vessel complications. *Journal of Pediatric Endocrinology and Metabolism*.

[2] Järvisalo MJ, Raitakari M, Toikka JO (2004). Endothelial dysfunction and increased arterial intima-media thickness in children with type 1 diabetes. *Circulation*.

[3] Wiltshire EJ, Gent R, Hirte C, Pena A, Thomas DW, Couper JJ (2002). Endothelial dysfunction relates to folate status in children and adolescents with type 1 diabetes. *Diabetes*.

[4] Nadeau KJ, Regensteiner JG, Bauer TA (2010). Insulin resistance in adolescents with type 1 diabetes and its relationship to cardiovascular function. *Journal of Clinical Endocrinology and Metabolism*.

[5] Pena AS, Couper JJ, Harrington J (2012). Hypoglycemia, but not glucose variability, relates to vascular function in children with type 1 diabetes. *Diabetes Technology & Therapeutics*.

[6] Babar GS, Zidan H, Widlansky ME (2011). Impaired endothelial function in preadolescent children with type 1 diabetes. *Diabetes Care*.

[7] Yan J, Tie G, Park B, Yan Y, Nowicki PT, Messina LM (2009). Recovery from hind limb ischemia is less effective in type 2 than in type 1 diabetic mice: roles of endothelial nitric oxide synthase and endothelial progenitor cells. *Journal of Vascular Surgery*.

[8] Orlandi A, Chavakis E, Seeger F, Tjwa M, Zeiher AM, Dimmeler S (2010). Long-term diabetes impairs repopulation of hematopoietic progenitor cells and dysregulates the cytokine expression in the bone marrow microenvironment in mice. *Basic Research in Cardiology*.

[9] Kränkel N, Armstrong SP, McArdle CA, Dayan C, Madeddu P (2010). Distinct kinin-induced functions are altered in circulating cells of young type 1 diabetic patients. *PloS One*.

[10] Sibal L, Aldibbiat A, Agarwal SC (2009). Circulating endothelial progenitor cells, endothelial function, carotid intima-media thickness and circulating markers of endothelial dysfunction in people with type 1 diabetes without macrovascular disease or microalbuminuria. *Diabetologia*.

[11] van Oostrom O, de Kleijn DPV, Fledderus JO (2009). Folic acid supplementation normalizes the endothelial progenitor cell transcriptome of patients with type 1 diabetes: a case-control pilot study. *Cardiovascular Diabetology*.

[12] Dimeglio LA, Tosh A, Saha C (2010). Endothelial abnormalities in adolescents with type 1 diabetes: a biomarker for vascular sequelae?. *Journal of Pediatrics*.

[13] Cai H, Harrison DG (2000). Endothelial dysfunction in cardiovascular diseases: the role of oxidant stress. *Circulation Research*.

[14] Stehouwer CDA, Lambert J, Donker AJM, van Hinsbergh VWM (1997). Endothelial dysfunction and pathogenesis of diabetic angiopathy. *Cardiovascular Research*.

[15] Hoffman RP, Hausberg M, Sinkey CA, Anderson EA (1999). Hyperglycemia without hyperinsulinemia produces both sympathetic neural activation and vasodilation in normal humans. *Journal of Diabetes and its Complications*.

[16] Dye AS, Huang H, Bauer JA, Hoffman RP (2012). Hyperglycemia increases muscle blood flow and alters endothelial function in adolescents with type 1 diabetes. *Experimental Diabetes Research*.

[17] Hirsch IB, Brownlee M (2005). Should minimal blood glucose variability become the gold standard of glycemic control?. *Journal of Diabetes and its Complications*.

[18] Ceriello A, Esposito K, Piconi L (2008). Oscillating glucose is more deleterious to endothelial function and oxidative stress than mean glucose in normal and type 2 diabetic patients. *Diabetes*.

[19] Higashi Y, Yoshizumi M (2003). New methods of evaluate endothelial function: method for assessing endothelial function in humans using a strain-gauge plethysmography: nitric oxide-dependent and -independent vasodilation. *Journal of Pharmacological Sciences*.

[20] Fayh AP, Krause M, Rodrigues-Krause J (2013). Effects of L-arginine supplementation on blood flow, oxidative stress status and exercise responses in young adults with uncomplicated type I diabetes. *European Journal of Nutrition*.

[21] van Gurp PJ, Lenders JWM, Tack CJ (2007). Increased forearm blood flow in longstanding Type 1 diabetic patients without microvascular complications. *Diabetic Medicine*.

[22] Berenson GS, Srinivasan SR, Nicklas TA (1998). Atherosclerosis: a nutritional disease of childhood. *American Journal of Cardiology*.

[23] Furumoto T, Saito N, Dong J, Mikami T, Fujii S, Kitabatake A (2002). Association of cardiovascular risk factors and endothelial dysfunction in Japanese hypertensive patients: implications for early atherosclerosis. *Hypertension Research*.

[24] Cé GV, Rohde LE, da Silva AMV, Puñales MK, de Castro AC, Bertoluci MC (2011). Endothelial dysfunction is related to poor glycemic control in adolescents with type 1 diabetes under 5 years of disease: evidence of metabolic memory. *Journal of Clinical Endocrinology and Metabolism*.

[25] Duck MM, Hoffman RP (2007). Impaired endothelial function in healthy African-American adolescents compared with caucasians. *Journal of Pediatrics*.

[26] Hortenhuber T, Rami-Mehar B, Satler M (2013). Endothelial progenitor cells are related to glycemic control in children with type 1 diabetes mellitus over time. *Diabetes Care*.

